# Supplementary material for: C/EBPβ Coupled with E2F2 Promoted the Proliferation of hESC-Derived Hepatocytes through Direct Binding to the Promoter Regions of Cell-Cycle-Related Genes
Source: Cells. 2023 Feb 2;12(3):497. doi: 10.3390/cells12030497 (PMC9914899; doi:10.3390/cells12030497)
Supplement: Supplementary file 1 [file cells-12-00497-s001.zip › cells-2085167-supplementary.pdf]

cells

MDPI

*Supplementary Materials*

# C/EBPβ Coupled with E2F2 Promoted the Proliferation of hESC-Derived Hepatocytes through Direct Binding to the Pro-moter Regions of Cell-Cycle-Related Genes

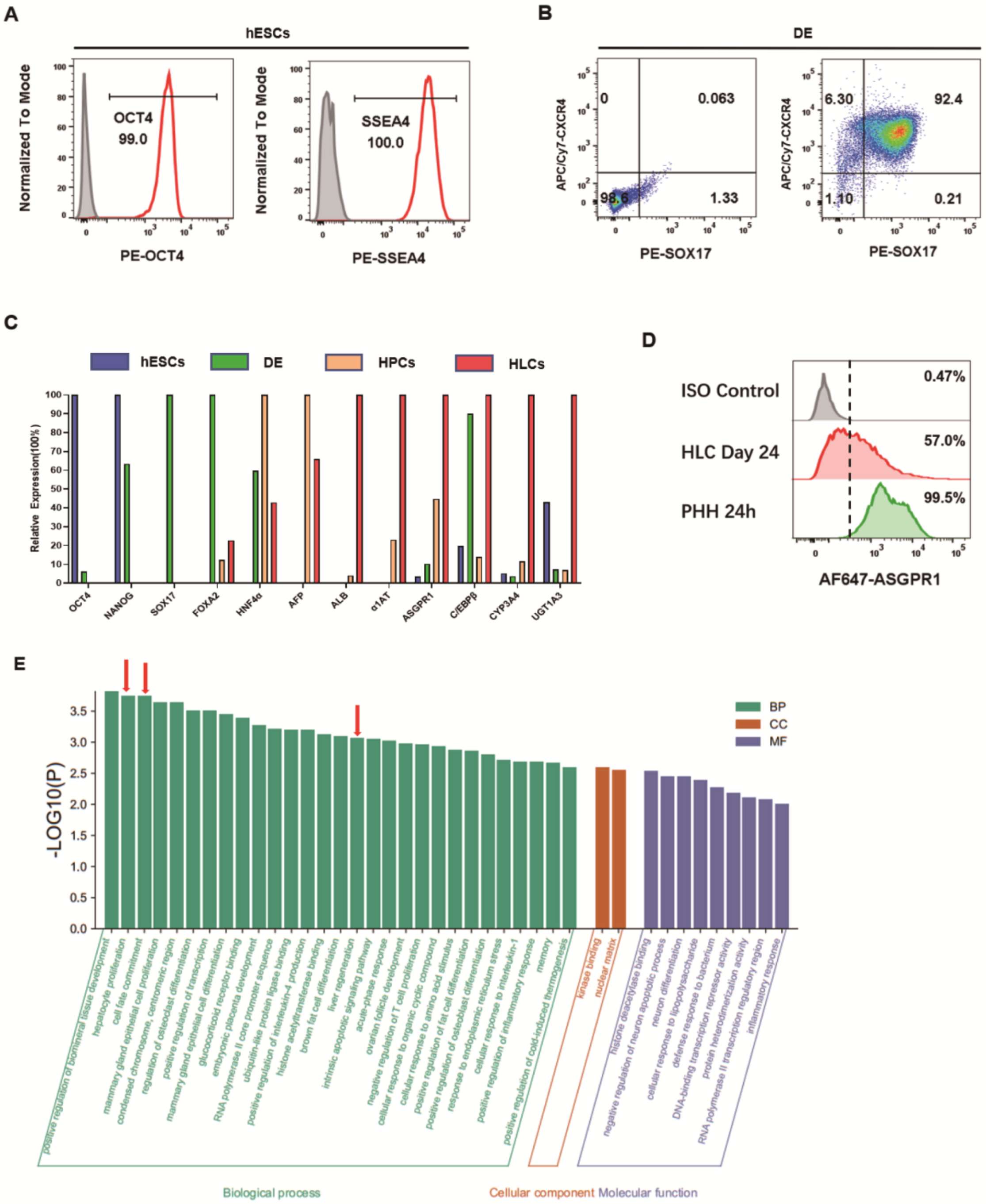


F

**Figure S1.** C/EBPβ was expressed in hepatocytes and involved in hepatocyte differentiation. (**A**,**B**) Flow cytometry analysis for the expression of specific markers at different stages of hESCs (OCT4, SSEA-4), DE cells (SOX17, CXCR4). (**C**) The relative expression of specific markers at different stages during the differentiation was determined by qPCR. (**D**) Flow cytometry analysis for the expression of mature hepatocyte marker ASGPR1. (**E**) GO terms were statistically and significantly associated

with the input gene C/EBPβ, and enrichment analyses were available by using the online website KOBAS-intelligence.

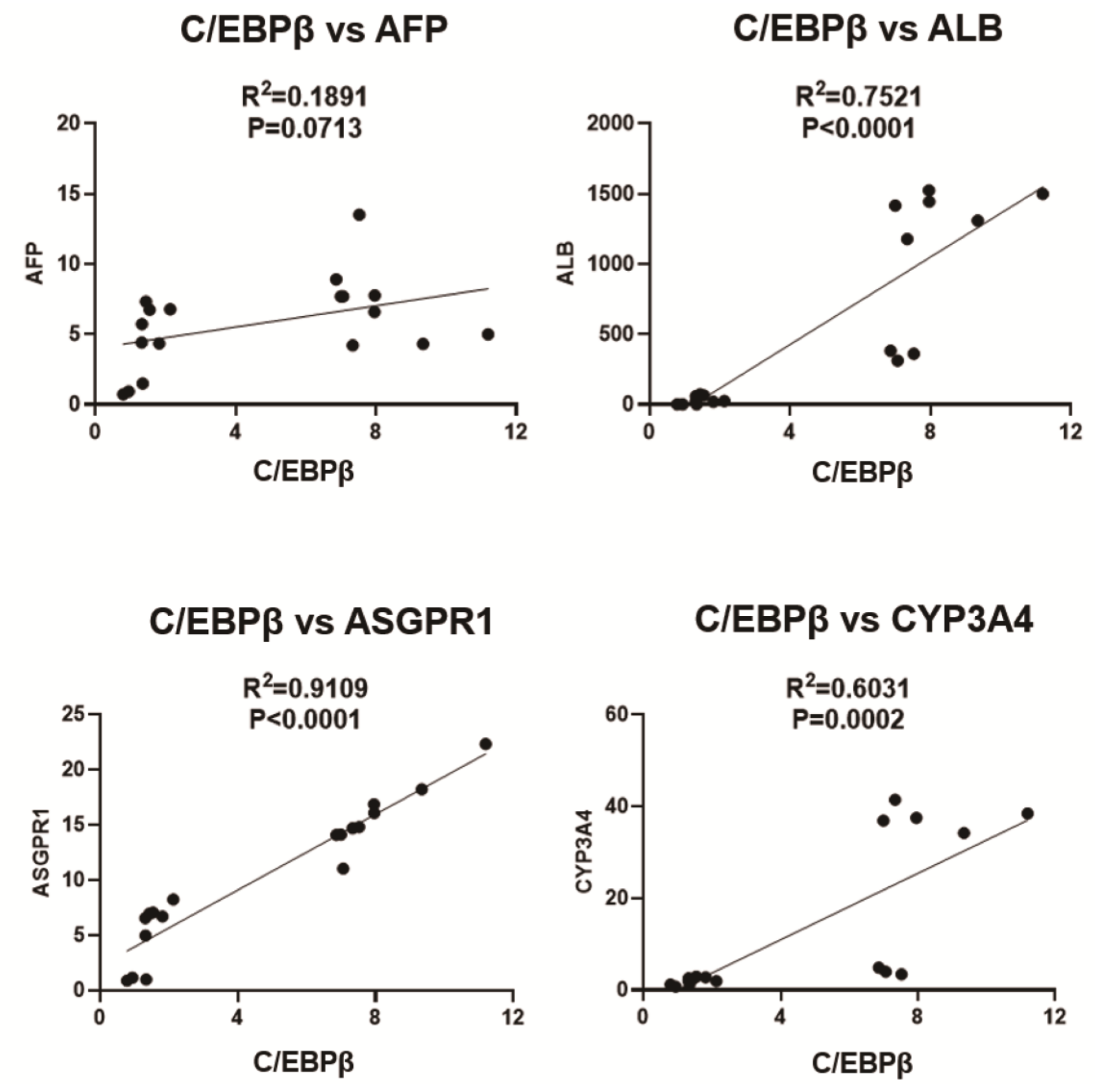


**Figure S2.** Correlation analysis of C/EBPβ gene expression with AFP, ALB, ASGPR1 and CYP3A4 during the differentiation of hepatocyte-like cells (HLCs) from hESCs.

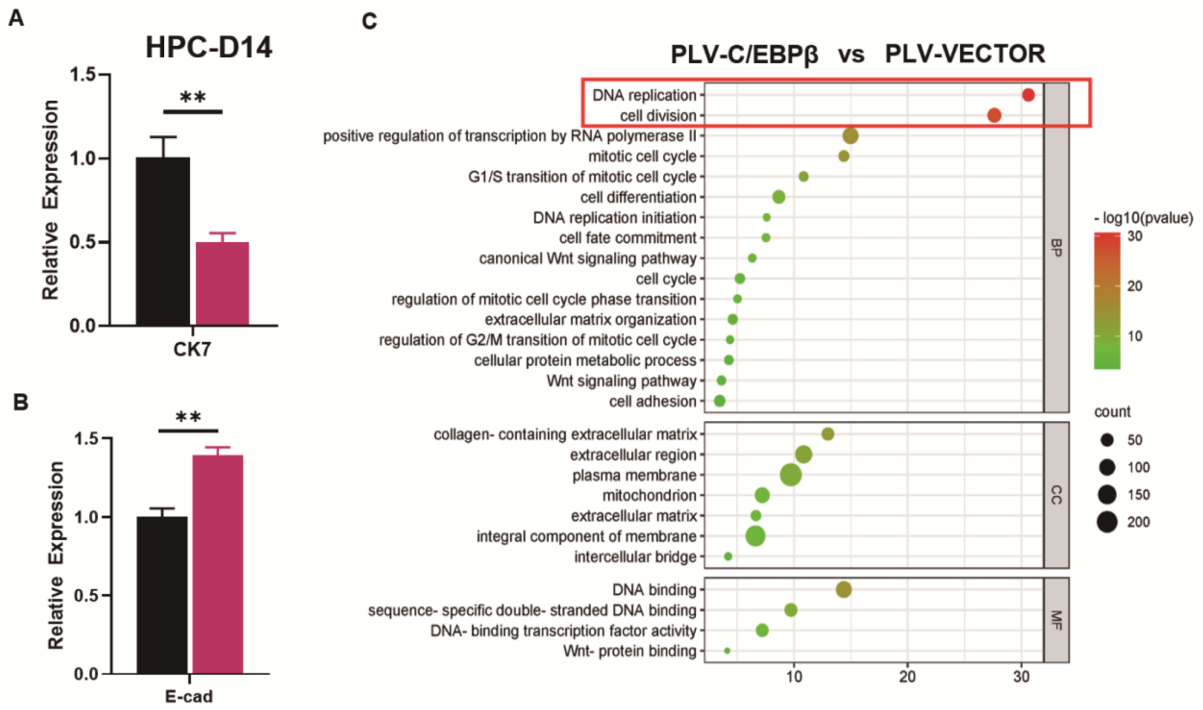


**Figure S3.** Enhanced expression of C/EBPβ promoted hepatic differentiation and cell cycle progression in HPCs. (**A,B**) Relative expression analysis of bile duct gene CK7 (**A**) and epithelia marker E-cad (**B**) were determined by qPCR. (**C**) GO analysis of the differentially expressed genes (DEGs), and the GO categories included BP and CC as well as MF respectively. Highly enriched GO terms showed that DNA replication and cell division were ranked at the top in the lists.

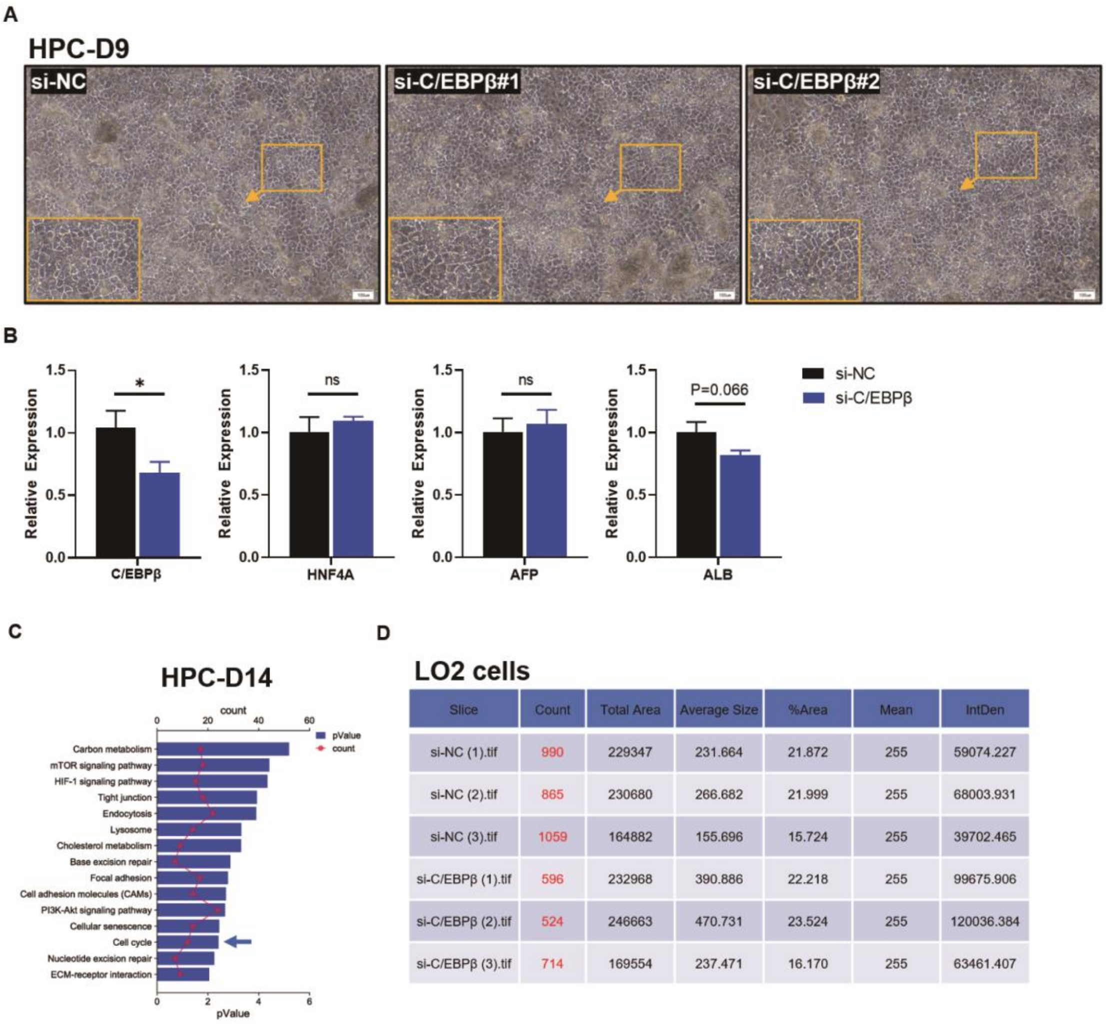


| Slice | Count | Total Area | Average Size | %Area | Mean | IntDen |
|---|---|---|---|---|---|---|
| si-NC (1).tif | 990 | 229347 | 231.664 | 21.872 | 255 | 59074.227 |
| si-NC (2).tif | 865 | 230680 | 266.682 | 21.999 | 255 | 68003.931 |
| si-NC (3).tif | 1059 | 164882 | 155.696 | 15.724 | 255 | 39702.465 |
| si-C/EBPβ (1).tif | 596 | 232968 | 390.886 | 22.218 | 255 | 99675.906 |
| si-C/EBPβ (2).tif | 524 | 246663 | 470.731 | 23.524 | 255 | 120036.384 |
| si-C/EBPβ (3).tif | 714 | 169554 | 237.471 | 16.170 | 255 | 63461.407 |

**Figure S4.** C/EBPβ Knockdown at the early stage had little impact on the hepatic differentiation from hESCs. (**A**) Representative images of cell morphologies 2 days after the treatment with 2 pairs of si-RNA at day 7 during the hepatic differentiation. (**B**) The expression of transcription factors (C/EBPβ and HNF4A) and hepatocyte genes (AFP and ALB) were detected by qRT-PCR at day 9 after the treatment with siRNAs at day 7. (**C**) The KEGG pathways for the differentially expressed genes in HPCs treated with si-NC or with si-C/EBPβ at day 14. (**D**) si-RNA mediated C/EBPβ knockdown were conducted in LO2 cells, and DAPI staining was performed, then cell numbers were defined by ImageJ software.

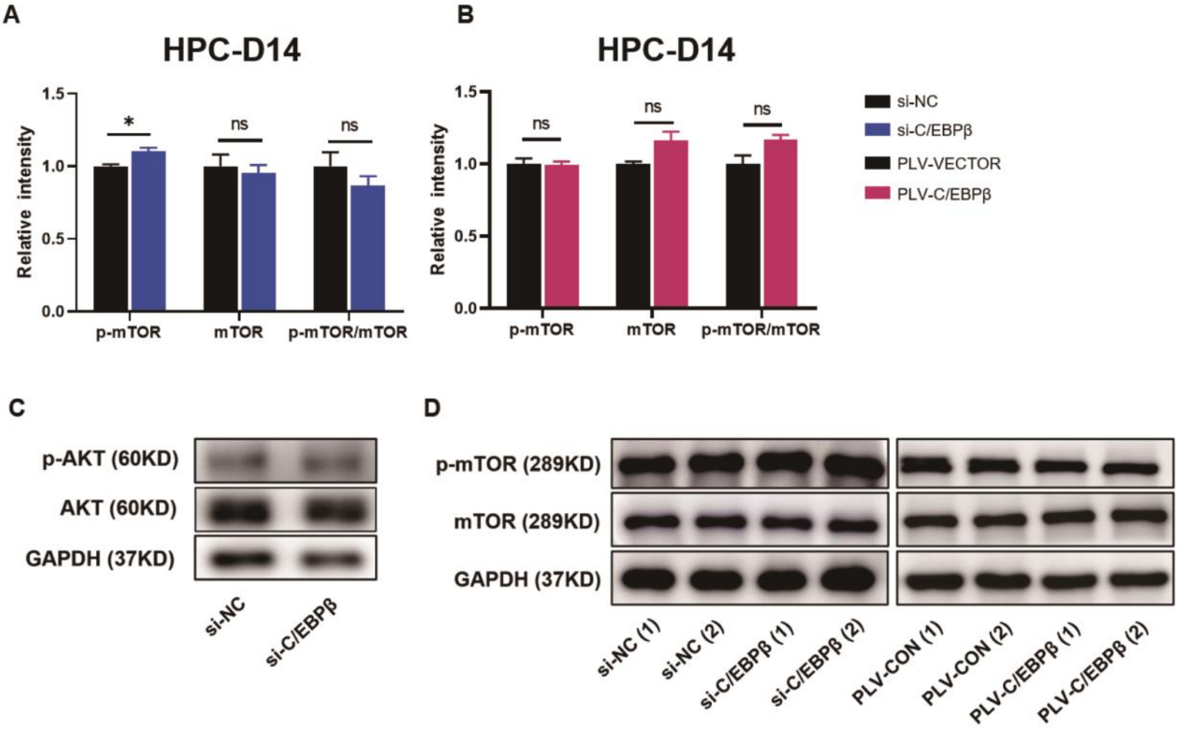


**Figure S5.** C/EBPβ did not orchestrate the PI3K/AKT/mTOR signaling pathway to promote cell proliferation. (**A,B**) The statistical results of mTOR/p/mTOR protein expressions in Fig. S5D. (**C**) Western blot analysis of the AKT/p-AKT after the knockdown of C/EBPβ at day 14 of HPCs. (**D**) Western blot analysis of the PI3K/AKT/mTOR signaling pathway in knockdown (left panel) or overexpression (right panel) of C/EBPβ on day 14 of HPCs.

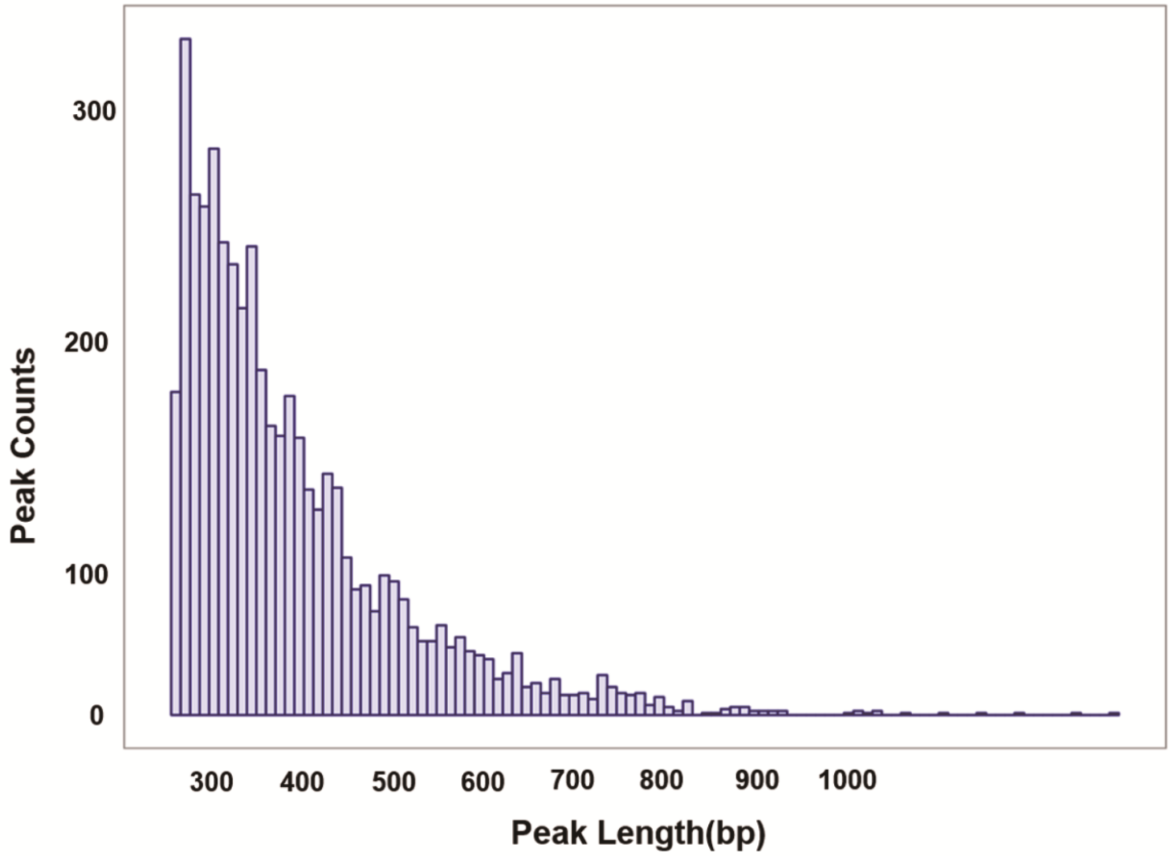

**Figure S6.** Length distribution of peaks in Cut&Tag analysis for efficient C/EBPβ binding, the abscissa is the length of peaks (bp), and the ordinate is the number of corresponding peaks.

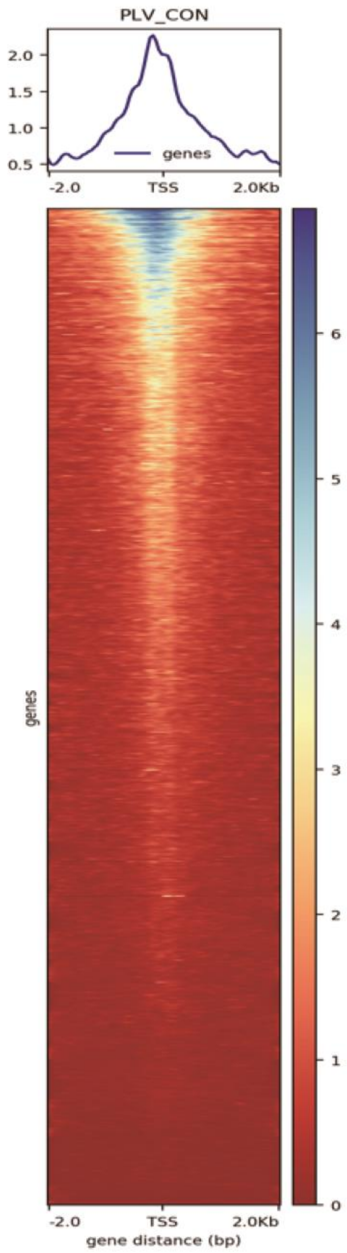


**Figure S7.** Heat map around genomic sites called from C/EBPβ binding profile in Cut&Tag analysis for efficient C/EBPβ binding.

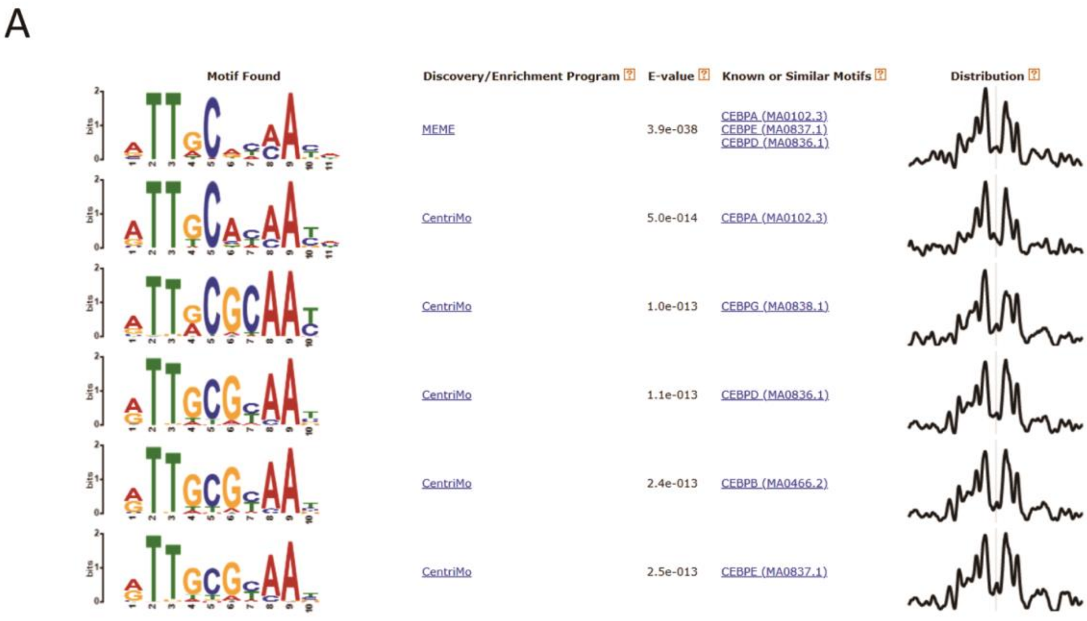


B

| | | | | | |
|---|---|---|---|---|---|
| 11 | Cell cycle | ko04110 | K02091 | ENSG00000105810 | CDK6; cyclin-dependent kinase 6 [EC:2.7.11.22] |
| | | | K02178 | ENSG00000169679 | BUB1; checkpoint serine/threonine-protein kinase [EC:2.7.11.1] |
| | | | K02214 | ENSG00000097046 | CDC7; cell division control protein 7 [EC:2.7.11.1] |
| | | | K02542 | ENSG00000076003 | MCM6; DNA replication licensing factor MCM6 [EC:3.6.4.12] |
| | | | K02603 | ENSG00000085840 | ORC1; origin recognition complex subunit 1 |
| | | | K03083 | ENSG00000082701 | GSK3B; glycogen synthase kinase 3 beta [EC:2.7.11.26] |
| | | | K03350 | ENSG00000004897 | APC3, CDC27; anaphase-promoting complex subunit 3 |
| | | | K03354 | ENSG00000196510 | APC7; anaphase-promoting complex subunit 7 |
| | | | K03364 | ENSG00000105325 | CDH1; cell division cycle 20-like protein 1, cofactor of APC complex |
| | | | K03868 | ENSG00000100387 | RBX1, ROC1; RING-box protein 1 [EC:2.3.2.32] |
| | | | K04402 | ENSG00000116717 | GADD45; growth arrest and DNA-damage-inducible protein |
| | | | K04500 | ENSG00000175387 | SMAD2; mothers against decapentaplegic homolog 2 |
| | | | K04728 | ENSG00000149311 | ATM, TEL1; serine-protein kinase ATM [EC:2.7.11.1] |
| | | | K04802 | ENSG00000132646 | PCNA; proliferating cell nuclear antigen |
| | | | K06619 | ENSG00000097007 | ABL1; abelson tyrosine-protein kinase 1 [EC:2.7.10.2] |
| | | | K06627 | ENSG00000145386 | CCNA; cyclin-A |
| | | | K06628 | ENSG00000093009 | CDC45; cell division control protein 45 |
| | | | K06636 | ENSG00000072501 | SMC1; structural maintenance of chromosome 1 |
| | | | K06639 | ENSG00000079335 | CDC14; cell division cycle 14 [EC:3.1.3.16 3.1.3.48] |
| | | | K06641 | ENSG00000183765 | CHEK2; serine/threonine-protein kinase CHEK2 [EC:2.7.11.1] |
| | | | K06669 | ENSG00000108055 | SMC3, CSPG6; structural maintenance of chromosome 3 (chondroitin sulfate proteoglycan 6) |
| | | | K08866 | ENSG00000112742 | TTK, MPS1; serine/threonine-protein kinase TTK/MPS1 [EC:2.7.12.1] |
| | | | K09389 | ENSG00000007968 | E2F2; transcription factor E2F2 |
| | | | K09392 | ENSG00000114126 | TFDP2; transcription factor Dp-2 |
| | | | K10152 | ENSG00000112576 | CCND3; G1/S-specific cyclin-D3 |
| | | | K16197 | ENSG00000164924 | YWHAB_Q_Z; 14-3-3 protein beta/theta/zeta |
| | | | K16198 | ENSG00000170027 | YWHAG_H; 14-3-3 protein gamma/eta |
| | | | K25228 | ENSG00000110200 | |

**Figure S8**. Cut&Tag analysis for efficient C/EBPβ binding. (**A**) The top 6 motif sequences associated with C/EBPβ binding showed high similarity to known protein-binding motifs in CEBP family. (**B**) Gene lists from KEGG enrichment in Fig. 6D, which showed that cell cycle signaling pathway was significantly enriched.

**Table S1.** The antibodies used for Flow cytometry in this study.

| Antigen | Cojugate | Company | Catalog number | Clone |
|---|---|---|---|---|
| OCT4 | PE | STEMCELL | 60093PE | 3A2A20 |
| TRA-1-81 | PE | STEMCELL | 60065PE | TRA-1-81 |
| CXCR4 | APC/Cy7 | Biolegend | 306528 | 12G5 |
| SOX17 | PE | BD Pharmingen | 561591 | P7-969 |
| AFP | PE | BD Pharmingen | 563002 | C3/AFP |
| ALB | PE | R&D | IC1455P | / |
| $\alpha$1-AT | FITC | Bethyl | A80-122F | / |
| Isotype (IgG1,κ) | PE | BD Pharmingen | 556650 | / |
| Isotype (IgG1,κ) | APC/Cy7 | BD Pharmingen | 557873 | / |
| Isotype (IgG2b,κ) | PE | BD Pharmingen | 555742 | / |

**Table S2.** The primers used for real-time quantitative PCR in this study.

| Name | Primers (Forword ; 5′ to 3′) | Primers (Reverse ; 5′ to 3′) |
|---|---|---|
| C/EBPβ | GCCCTCGCAGGTCAAGAGCA | TTGAACAAGTTCCGCAGGGTG |
| C/EBPβ (EXO) | CGACGGAGAC-TACAAGGATCATGA | TTGCCGCCGTACGCAG-CAGCCAA |
| C/EBPα | CTCGAGGCTTGCCAGACCGT | GCGGGCTTGTCGGGATCTCAG |
| ALB | GAGACCAGAGGTTGATGTGATG | AGTTCCGGGG-CATAAAAGTAAG |
| AFP | GGGAGCGGCTGACATTAT | TGTTTCATCCACCACCAA |
| α1-AT | TCGCTACAGCCTTTGCAATG | TTGAGGGTACGGAGGAGTTCC |
| ASGPR1 | GAAGCAGTTCGTGTCTGACCTG | AGCGAGAGAACCAGTAG-CAGCT |
| HNF4α | GGTGTCCATACGCATCCTTGAC | AGCCGCTTGATCTTCCCTGGAT |
| CYP3A7 | AAGGGCTATTGGACGTTTGACA | ATCCCACTGGCCCGAAAG |
| CK7 | TCCGCGAGGTCACCATTAAC | GCTCTGTCAACTCCGTCTCAT |
| CK19 | ACCAAGTTTGAGACGGAACAG | CCCTCAGCGTACTGATTTCCT |
| CDC25C | AGAAGCCCATCGTCCCTTTGGA | GCAGGATACTGGTTCAGAGACC |
| CDC45L | TGGATGCTGTCCAAGGACCTGA | CAGGACACCAACATCAG-TCACG |
| PCNA | CAAGTAATGTCGATAAAGAG-GAGG | GTGTCACCGTTGAAGAGAG-TGG |
| E2F1 | GGACCTGGAAACTGACCATCAG | CAGTGAGGTCTCATAGCGTGAC |
| E2F2 | CTCTCTGAGCTTCAAGCACCTG | CTTGACGGCAATCACTGTCTGC |
| MCM3 | CGAGACCTAGAAAATGGCAGCC | GCAGTGCAAAGCACAT-ACCGCA |
| GINS1 | GCAAAGTCAGGTGGAC-GAAGTG | CTGATCCGAAGCAAGCGGTCAT |
| GAPDH | GAAGATGGTGATGGGATTTC | GAAGGTGAAGGTCGGAGTC |

**Table S3.** The antibodies used for Western Blot in this study.

| Antigen | Species Reactivity | Company | Catalog number | Dilution |
|---|---|---|---|---|
| C/EBPβ | Mouse anti-human | Santa Cruz | sc-7962X | 1:1000 |
| CDC45L | Rabbit pAb | GeneTex | GTX110586 | 1:1000 |
| PCNA | Rabbit pAb | GeneTex | GTX100539 | 1:1000 |
| E2F2 | Mouse anti-human | Santa Cruz | sc-9967 | 1:500 |
| AKT(pan) | Rabbit pAb | CST | 4691T | 1:1000 |
| p-AKT(T308) | Rabbit pAb | CST | 13038T | 1:1000 |
| mTOR | Rabbit pAb | CST | 2983T | 1:1000 |
| p-mTOR | Rabbit pAb | CST | 5536T | 1:1000 |
| CyclinD1 | Rabbit pAb | CST | 55506T | 1:1000 |
| E-cad | Rabbit pAb | CST | 3195S | 1:1000 |
| GAPDH | Rabbit pAb | GeneTex | GTX100118 | 1:5000 |
| IgG (H+L) | Goat anti-mouse | Beyotime | A0216 | 1:2000 |
| IgG (H+L) | Goat anti-rabbit | Beyotime | A0208 | 1:2000 |

**Table S4.** The siRNA(C/EBPβ) used in this study.

| Oligo Name | Primer | Sequence (5′-3′) |
|---|---|---|
| hs-C/EBPβ-si-1 | F′ | CCUGCCUUUAAAUCCAUGGdTdT |
| | R′ | CCAUGGAUUUAAAGGCAGGdTdT |
| hs-C/EBPβ-si-2 | F′ | CGACUUCCUCUCCGACCUCdTdT |

| | | |
|---|---|---|
| | R′ | GAGGUCGGAGAGGAAGUCGdTdT |
| hs-C/EBPβ-si-3 | F′ | GCACAGCGACGAGUACAAGdTdT |
| | R′ | CUUGUACUCGUCGCUGUGCdTdT |
| si-NC | F′ | UUCUCCGAACGUGUCACGUdTdT |
| | R′ | ACGUGACACGUUCGGAGAAdTdT |

**Table S5.** The shRNA(C/EBPβ) target sequences in this study.

| Name | Symbol | Location | Target Sequence |
|---|---|---|---|
| sh-con | / | / | ACAGAAGCGATTGTTGATC |
| sh-a | C/EBPβ | 505 | GCCTGCCTTTAAATCCATGGA |
| sh-b | C/EBPβ | 1258 | GCACAGCGACGAGTACAAGAT |
| sh-c | C/EBPβ | 1432 | GCGGAACTTGTTCAAGCAGCT |
